# Meteorological Register for September, 1817

**Published:** 1817-11

**Authors:** John Bailey

**Affiliations:** Surgeon, &c.


### Meteorological Register for September, 1817,

*Kept at Harwich, in Essex,*

By JOHN BAILEY, Surgeon, &c.

| | Moon's Age. | Atmospherical Pressure. Morn. | Ev. | Temp. | Wind. | Remarks. | General Statement of Diseases. |
|---|---|---|---|---|---|---|---|
| | 1 | 29 8½ | 29 8½ | 67 | W | Fine and clear | Cholera |
| | 2 | 30 | 30 | 66 | W | ditto | |
| ☾ | 3 | 29 9 | 29 9 | 67 | E | ditto | Rubeola |
| | 4 | 9 | 30 | 73 | N | ditto | |
| | 5 | 30 ½ | ½ | 70 | NW | ditto | |
| | 6 | | | 69 | E | ditto | |
| | 7 | | | 67 | E | ditto | |
| | 8 | 29 9½ | 29 9½ | 68 | E | ditto | |
| | 9 | | 30 | 72 | E | ditto | |
| | 10 | 30 | | 67 | NE | ditto | |
| ● | 11 | | | 66 | E | ditto | |
| | 12 | 29 9 | 29 9 | 65 | W | Rain | |
| | 13 | 9½ | 9½ | 64 | E | Cloudy | |
| | 14 | 9 | 9 | 64 | NE | Rain | |
| | 15 | 9½ | 9½ | 66 | E | Rain. Windy | |
| | 16 | 30 | 30 | 64 | S | ditto | |
| ☽ | 17 | | 29 4 | 65 | NE | ditto | |
| | 18 | 7 | 6½ | 61 | NE | Rain | |
| | 19 | 7½ | 3½ | 65 | W | ditto | |
| | 20 | 9 | 9 | 65 | NW | | |
| | 21 | | | 63 | N | | |
| | 22 | 8½ | 8 | 61 | NW | Rain | |
| | 23 | 8 | 8½ | 64 | NW | | |
| | 24 | 8½ | | 63 | S | | |
| ○ | 25 | 6½ | 4 | 65 | W | Rain. Windy | |
| | 26 | 2½ | 2½ | 63 | W | R. Gale of Wind | |
| | 27 | 3 | 3 | 63 | SW | Windy | |
| | 28 | 6 | 8 | 57 | SW | | |
| | 29 | 9 | 9 | 57 | NW | | |
| | 30 | | | 55 | E | | |

☞ Authors and Publishers are requested to send Copies of new Works for Review as early as possible after their publication. They will be reviewed early in proportion.

